# Transforming Biomedical Uncertainty: the socio-historical origins of myalgic encephalomyelitis (ME) and chronic fatigue syndrome (CFS)

**DOI:** 10.1136/medhum-2025-013657

**Published:** 2026-08-14

**Authors:** Sally Cross

**Affiliations:** Centre for the History of Science, Technology and Medicine, The University of Manchester, Manchester, UK

## Abstract

Myalgic encephalomyelitis (ME) and chronic fatigue syndrome (CFS) are serious and disabling long-term conditions characterised by uncertainty surrounding their aetiology, diagnosis and treatment. People with ME/CFS struggle to be understood, taken seriously, and supported with their illness. At least since the 1990s, ME and CFS have been considered by many to be related, overlapping or synonymous with one another. However, the concepts originated from different socio-historical contexts. This article disentangles the histories of ME and CFS, roots them in the United Kingdom and the United States respectively, and compares how they emerged as medical and scientific objects. Drawing on a critical literature analysis of medical texts between 1950 and 1990, I explore how both ME and CFS materialised through the regulation of uncertainty within biomedical systems. In both cases, uncertainty was transformed into knowledge by systematically obscuring certain aspects of illness. These transformations shaped what could be known about these conditions in the decades to come and may be at the root of the epistemic injustices experienced by patients. Those who campaign for more scientific research into ME/CFS should be wary of the propensity for biomedicine to generate ignorance in the face of these complex conditions. This analysis contributes to a growing body of research on the medical sociology of ignorance, further illustrating the value of uncertainty and ignorance as heuristic tools for understanding the politics of knowledge production within biomedical systems.

## Introduction

Myalgic encephalomyelitis (ME) and chronic fatigue syndrome (CFS) are serious and disabling long-term conditions characterised by uncertainty regarding their aetiology, diagnosis and treatment. Presently, in United Kingdom (UK) and United States (US) health policy ME and CFS are coupled as ‘ME/CFS’ and described as a symptom complex of fatigue, post-exertional malaise (PEM)^1^, unrefreshing sleep and cognitive impairment; ME/CFS is considered to be more common in women than men [1,2].

Some of the most salient histories of ME/CFS were published in the 1990s by historian Edward Shorter [3] and journalist Elaine Showalter [4], both of whom argued that the illness is, and always has been, psychosomatic: essentially a contemporary renaming of hysteria. The term ‘psychosomatic’ has a long history of stigmatising patients who experience medically uncertain conditions. As explored in a special issue of *Medical Humanities* [5], it is also a highly ambiguous term. Following those who have considered the social construction of knowledge concerning ME and CFS [6–10], I situate different versions of the condition in their particular socio-historical contexts. Rather than presenting ME/CFS as fundamentally a psychosomatic or organic disease, I draw attention to the multiplicity of knowledge that arises from categorising medical uncertainty. Other historical accounts have focused on the emergence of CFS in the US [6–9], or traced broad shifts in medical conceptualisations of ‘long-term exhaustion’ [10]. In this article, I contribute to scholarship on ME/CFS by examining the introduction of ‘ME’ in the UK in 1956 and contrasting this to the emergence of ‘CFS’ in the US in 1988, thereby offering a comparative history of the two halves of ME/CFS. I also introduce a heuristic of ignorance to examine how forms of biomedical authority regulate, obscure and transform uncertainty surrounding these conditions.

This article explores how ME and CFS originated as objects of scientific research and medical practice. I draw from a sociology of diagnosis, which considers how subjective experiences are transformed into medical syndromes [11]. In his influential book *Framing Disease: Illness, Society, and History*, the medical historian Charles E. Rosenberg [12] described how diseases come into being when we agree on how they should be named and framed. Similarly, the physician and medical historian Robert Aronowitz [13] emphasised that whilst medical classifications take into account biological and clinical factors, they are predominantly shaped by social negotiations; symptoms can be transformed into disease even in the absence of biomedical data. Whilst there are legitimate claims to a longer history of ME/CFS [see e.g., 14], I am interested in the particular origins of ME and CFS as names, concepts and objects of biomedical research.

Aronowitz [6] identified that whilst patient groups have long been strong advocates for the rights of people with ME/CFS to healthcare, they have rarely critiqued the reliance of these rights on legitimacy within a biomedical model. ‘Radical epistemological critiques of biomedical models have been rare. Biomedical failure to confirm these syndromes as diseases is seen as temporary, provisional, and correctable by further research’ [6, p36]. Responding to Aronowitz’s observation, this article draws on the sociology of ignorance to emphasise how diagnostic categories regulate uncertainty, and, in doing so, generate both knowledge and ignorance [15]. Research on the social production of ignorance has proliferated over the past two decades, producing a range of concepts for analysing the unknown [see 16]. I focus on the emergence of ‘uncomfortable knowledge’ [17], ‘undone science’ [18] and ‘strategic unknowns’ [19–21] in relation to ME/CFS. Central to my argument is a conceptual distinction between *uncertainty*, which reflects complex yet potentially knowable phenomena (e.g., subjective experiences of pain), and *ignorance*, in which forms of knowledge are made absent or unintelligible. I argue that *known unknowns* surrounding the events leading to the conceptualisation of ME and CFS were obscured through the formation of these diagnostic categories, ultimately shaping how the conditions were understood and investigated. In this way, I explore how uncertainty was transformed into ignorance.

People with ME/CFS have long struggled to have their experiences recognised as legitimate and to access healthcare and state support [6,22–24]. Advocacy organisations also frequently draw attention to the limited funding allocated for research and specialist care for the condition [see e.g., 25]. Fricker’s [26] concept of ‘epistemic injustice’, or injustice relating to knowledge, describes both the experience of having few conceptual resources to understand yourself, and belonging to a group whose expertise is not considered credible. Fricker argues that medically unexplained conditions are an example of ‘bad luck’ rather than injustice [26, p152], a distinction arrived at by sidelining the power relations that shape disability and illness [27]. Nonetheless, several researchers have utilised the concept of epistemic injustice to describe how people with ME/CFS are harmed by those who question the legitimacy of their condition [28–31]. As Byrne [32] identifies, this research largely focuses on clinical encounters, and at times reduces epistemic injustice to professionals harbouring negative stereotypes of patients. In this article, I provide historical context to consider why *both* patients and medical professionals can struggle to make sense of ME/CFS. I frame epistemic injustice as a structural inequality that cannot ‘be prevented by simply confronting the attitudes of the medical professionals in question’ [32, p375]. This article considers ignorance not as the result of the prejudice of medical professionals but as something that constitutes it, taking what Bain describes as an ‘ignorance-first approach’ [33, p47].

This article explores the socio-historical origins of ME and CFS, comparing how they originated as biomedical objects. My analysis of biomedical literature roots ME and CFS in the UK and the US, respectively, setting up this article as a journey across the Atlantic. The origins of ME will take us to an outbreak of illness at Royal Free Hospital, London, in 1955, and then to its reimagining by two clinicians in 1970. To understand how CFS originated, after some consideration of its rootlessness, we will travel to Incline Village on the north shore of Lake Tahoe, Nevada, in the Winter of 1984-85. In both of these cases, I show how the medical concepts materialised as a means to regulate uncertainty within biomedical systems and explore the potential consequences of this for the knowledge and legitimacy of ME/CFS in the decades that followed. ME and CFS both originated through a transformation of biomedical uncertainty, but the socio-historical contexts where this occurred are distinct, and so too are the actors that negotiated these transformations. ME is the product of debates between a medical community in the UK, including physicians, journals and patient groups, who shared both geographic location and epistemic values. In contrast, CFS was created as a means for a national public health agency to regulate incompatible claims to knowledge across the US’s large and complex medical system.

## Method^2^

This article is based on a critical analysis of the biomedical literature that first described ME and CFS as objects of concern for public health, clinical practice and scientific research. I identified literature using PubMed between April and July 2024. Initially, I used the search terms “myalgic encephalomyelitis”[Title] and “chronic fatigue”[Title], filtering for responses with abstracts available on PubMed. I read and analysed all abstracts and selected documents to be analysed in full based on their historical significance, discerned by a high number of citations, introduction of terminology or diagnostic criteria, and reports of key events. I also analysed some texts that appeared to be outliers to the wider medical discourse. I noted alternative nomenclature and conducted a second round of searches for these via PubMed. All search terms are summarised in Table 1 below. Other relevant literature was identified through the bibliographies of articles I analysed. I included literature from when the search terms first appeared in medical literature until 1990 (inclusive). In 1990, the concepts of ME and CFS became entangled, and researchers from the US and the UK came together to share knowledge [see 34].

I noted the research disciplines, country, journal, subject, and methods for all biomedical documents, and I kept notes of themes that emerged from comparing texts. By the end of the analysis, I had reviewed the abstracts of 118 documents, 25 of which I analysed in full. Table 2 below summarises some features of these documents. My analysis was both interpretive and systematic in that my findings emerged through iterative comparisons, whilst maintaining clear audit trials [35]. My reconstruction of medical debates was supported with analysis of documents from the media and patient groups, identified thanks to patient-led websites, including MEpedia.org and the Royal Free 1955 archive available on X (@RFH1955). For the analysis of CFS, context for medical documents was also gained through a reading of *Osler’s Web*, a journalistic account of events relating to CFS from 1984-1994 [36]. As I read the texts, I focused on language, categories, methodological tools and images, and how they were employed to justify claims of aetiology, diagnosis, or the best treatment strategies for ME and CFS. I also considered what was absent from the texts, attending to both the production of knowledge and ignorance.

### The origins of myalgic encephalomyelitis

#### An unknown epidemic at the Royal Free Hospital

The term ‘benign myalgic encephalomyelitis’ was introduced as ‘a new clinical entity’ in a 1956 edition of *The Lancet* [37]. ‘Myalgic’ referred to muscle pain, ‘encephalomyelitis’, inflammation of the spinal cord, and the condition was considered to have ‘a relatively benign outcome’ [37, p790]. The name was suggested as a means to group several recorded outbreaks of illness, which were characterised, in part, by muscular pains. These outbreaks had until that time been defined by either the location where they occurred, for example, ‘Iceland disease’ [38], or in their similarity to, but distinction from poliomyelitis, for example, ‘an illness resembling poliomyelitis’ [39]. Poliomyelitis was a matter of significant public concern in the post-war period, developing in parallel with visual media that spread images of people’s profound disablement [40]. However, it was an outbreak that began in July 1955 at the Royal Free Hospital in London and subsequent publications that brought together disparate literature on unexplained epidemics resembling poliomyelitis, drawing the attention of medical professionals in Britain.

Following an outbreak of illness at the Royal Free Hospital in 1955, several journal articles were published by medical professionals working at the hospital, foregrounded by Dr Melvin A. Ramsay [41–46]. They described how between 13^th^ July and 25^th^ July, 70 members of staff were affected by an ‘epidemic of highly infectious character’ [46]. The hospital closed between 25^th^ July and 5^th^ October, and by 24^th^ November, 292 members of staff had been affected, alongside only twelve patients. The condition was largely labelled ‘encephalomyelitis’ and the symptoms are described in detail, including pain, sensory disturbances, motor weakness, and muscle spasms. Crucially, researchers described both physical and psychological aspects of illness: ‘The earliest symptoms were usually malaise and headache, frequently associated with disproportionate depression and emotional lability.’ [46, p5050]. Ramsay and O’Sullivan described it as a ‘bizarre jumble of neurological signs and emotional disturbance’ [41, p1199].

It was challenging for clinicians based at the Royal Free Hospital to claim objective knowledge of this new clinical entity because they had not identified a physical agent: ‘In all the recorded outbreaks of cases […] the investigations have been hampered by the fact that there have been no deaths and no infective or toxic agent has been found [41, p764]’.^3^ This made it difficult to connect the wide-ranging symptoms to existing medical constructs (to convert symptoms to diagnosis), and in turn to emphasise the legitimacy of the phenomenon to the medical community. In the absence of a physical agent, researchers communicated the medical validity of Royal Free disease in alternative ways, particularly by using the language and signs associated with infectious diseases of known origin, thus entangling the uncertain phenomenon within this medical sphere.

Medical literature from other contexts that describes ‘outbreaks’ or ‘epidemics’ of illness with similar, but equally uncertain, characteristics was assembled to bolster the clinicians’ narratives. The paper that presented the collective findings of The Medical Staff of The Royal Free Hospital [46] references epidemics in Iceland in 1948-9 [47], Adelaide in 1949-51 [48], New York State in 1950 [38], the Middlesex Hospital, London, in 1952 [49], Coventry in 1953 [39], and Durban, South Africa, in 1955 [50]. These phenomena are all described as epidemics, i.e., as features of populations, not individuals. The Royal Free outbreak was also described in terms of infectious diseases, even if this was to distinguish it from them; for example, Ramsay [44] frequently emphasises that patients were ‘apyrexial’ (without fever). Similarly, as previously mentioned, many of these symptoms are described as ‘simulating poliomyelitis’, but the lack of markers in cerebrospinal fluid and findings from electromyographic studies meant that the conversion of these symptoms into a diagnosis of poliomyelitis was ‘untenable’ [41, p761]. The language used to describe this ‘new clinical entity’ is infectious, even if it is often utilised to distinguish the condition from other communicable diseases. Conditions defined by medical uncertainty are (quite obviously) difficult for medical practitioners to order within medical systems of knowledge. However, researchers at the Royal Free created order from chaos by utilising the tools and language of infectious diseases.

Clinicians presenting findings from the Royal Free outbreak referred to the epidemiological characteristics of the disease in terms of occupation and ‘sex’, but, perhaps unsurprisingly, did not explore how these factors affected illness: ‘Institutional life was an important factor in determining the case incidence which was highest among nurses, orderlies and resident domestic staff’ [43, p121]. Of the total hospital staff at risk of infection, 10.4% of women, compared to 2.8% of men, were reportedly affected by illness [46, p902]. Discussion of relations between disease and gender, or the social conditions of ‘institutional life’ as a nurse or domestic worker, is notably absent. In part, publications on different outbreaks were consolidated on the premise that they mostly affected nurses, but this is rarely explicitly addressed. Some reference was made to similarities between this new condition and ‘trench fever’ described in World War II [51], but clinicians at the Royal Free [46] distanced themselves from literature concerning soldiers impacted by ‘a disease resembling poliomyelitis’ [52]. In this way, ME was quietly feminised even as gender itself remained largely unexamined in medical discourse.

The dearth of experiential evidence, alongside the neglect of variables relating to sex/gender and occupation in scientific publications, makes it difficult to discern how the gendered working culture and environment of the Royal Free Hospital shaped the 1955 epidemic. Nevertheless, it is important to reflect on the experiences of nurses and domestic workers – including overwork, low pay and poor working conditions – and how these may have influenced the incidence and experience of illness. At the time, medical staff were adapting to changes in the health system following the formation of the NHS, there were significant pay disputes, and many migrant nurses were joining the workforce [53]. A richer history of Royal Free disease would therefore situate the experiences of nurses and domestic workers within the broader socio-political context of 1950s Britain, but such an analysis is beyond the scope of this article. A historical reconstruction of the Royal Free epidemic that centres gender, class (and intersecting oppressions) will need to move beyond what has been preserved in medical texts.

#### Royal Free epidemic: a reconsideration

In the 1950s and 60s, medical literature relating to ME was sparse, largely consisting of reports of outbreaks with similar characteristics to the Royal Free epidemic. In the documents I have analysed, ME as a diagnostic category does not appear to extend beyond the discourses of the small groups of clinicians who witnessed the epidemics, and of course, the lives of the people affected by them. It was not until 1970 that ME gained salience as a subject of wider medical and public debate. This arose from a paper that suggested a ‘reconsideration’ of the Royal Free epidemic [54]. The gendered characteristics of the epidemic had been largely ignored by physicians in the previous decades. In contrast, this new definition of ME placed femininity central to the disease.

Colin McEvedy and Alfred William Beard [54], psychiatrists based at Middlesex Hospital, London, reviewed the case notes of medical professionals involved in the Royal Free epidemic, fifteen years after the outbreak, and concluded that it was a case of ‘epidemic hysteria’. They framed hysteria as a psychological reaction to social segregation, particularly of young women, which manifests in a range of emotional and somatic symptoms. In an article in The British Medical Journal (BMJ), they disputed accounts of ME as an ‘organic disease’ related to ‘a viral infection of the central nervous system’ [54, p7]. Their justification rested on the ‘normal’ laboratory results, the dissociation between illness and fever, and its occurrence amongst ‘segregated females’ [54, p9]. The psychiatrists equated nurses’ segregation to that of ‘girls’ schools, convents and among female factory hands’ [54, p9], but they did not provide details of how nurses were segregated at the Royal Free Hospital. They did not explore alternative explanations as to why the illness mostly affected women, nor did they consider how an infectious disease may spread in a segregated population. Curiously, the psychiatrists also excluded some people (both men and women) affected by illness from their statistics, consequently increasing the proportion of women compared to men affected [54, p7].

In a second article in *The BMJ*, McEvedy and Beard reinforced their argument with evidence from other outbreaks, concluding that ‘a bona fide polio epidemic was the initiating stress for an hysterical response by the nursing community’ [55, p14]. Interestingly, they recognised how poliomyelitis ‘altered medical perception’, hinting at what has been discussed in this paper, that ME was shaped with and through the language of infectious disease [55, p13]. But the psychiatrists were equally unaware of how their medical perception shaped their understanding of ME. Despite the epidemic occurring just two miles from their workplace, they did not interview any of the people affected.

McEvedy and Beard’s ‘reconsideration’ [54] was culturally salient; it reframed ME as a matter worthy of discussion and debate. But it also cast doubt on ME’s existence as an organic disease. Although their paper dismissed previous understandings of ME and even the name itself, the debate sparked by their paper popularised ‘ME’ as a construct. Their argument was readily accepted by anglophone news media; the *Sunday Times* wrote: ‘The mystery of a strange new disease […] seems at long last to be solved’ [56]. *Time* magazine stated: ‘[ME] falls into the same category as the dancing manias of Germany in the Middle Ages or the Neapolitans’ tarantella’ [57]. McEvedy and Beard’s [54,55] framing of ME is both simple and sensational. It created a construction of ME that reaffirmed medicine’s authority over a phenomenon that had previously revealed the limits of medical knowledge. It dispelled uncertainty.

Medical professionals disputed McEvedy and Beard’s ‘reconsideration’ [54,55], and began to argue with greater certainty that ME was an organic disease. Physicians rebutted their findings through letters to *The BMJ*, for example Compston et al. [58] emphasised that they considered hysteria to be a valid diagnosis, but not in the case of the Royal Free Epidemic because of clinical evidence to the contrary. By 1975, several physicians, led by Melvin A. Ramsay, established a study group to encourage medical research on the condition [59], and in 1978 they published a special issue of the *Postgraduate Medical Journal* [60]. In this special issue, Compston [61] summarised the report of The Medical Staff of the Royal Free Hospital from 1957 [46] but focused more on physiological than emotional symptoms. Physicians also framed ME as a result of viruses endemic in the population to distinguish it from hysteria that was not thought to be endemic [62,63].

Throughout the 1970s and 80s, medical discourse became divisive between those who considered ME to be a hysterical reaction and those who considered it to be a (post-)viral disease. This polarised debate was shaped by an assemblage of UK physicians, patient groups and media organisations. The ME Association was founded to support patients and families, and promote an understanding of ME as a legitimate disease [64: cites 1976,65: cites 1980]. In 1986, an article in the *Observer* [66] presented ME as a serious and overlooked disease, sparking the ME Action Campaign (now Action for ME), but it also reignited voices in the media and medical establishment who argued ME was a disorder of femininity [67]. In 1987, the study group discussed above met again and reflected on the continued debate surrounding the Royal Free epidemic: ‘The division between those who believe that the outbreak was caused by an infection and those who favour a psychological explanation continues to echo down the years’ [59, p327].

By the late 1980s, ME was a condition characterised primarily by the controversy between those who framed it as psychosocial and those who argued it was an organic disease. In 1988, the BBC TWO documentary series *Horizon*, with average ratings of over two million throughout the eighties [68], aired an episode on ME featuring medical professionals and several people affected by the illness [69]. In this documentary, excerpts from interviews with McEvedy and Ramsay presented their infection vs. hysteria debate, and again, their arguments differed on whether there is enough physical evidence to take patients’ concerns seriously as an organic disease. McEvedy also described his reasoning for why women are more affected by ME: ‘the simple answer is they are much nicer than men, and they are much more sympathetic to other people’s problems’ [69, 19:55]. McEvedy reduced women to ‘females’ and entwined the feminine with the psychological. In the same year as the *Horizon* documentary, Ramsay published a book that charted the ‘saga of Royal Free Disease’ in which he described ME as (characterised by) ‘muscle tenderness’, ‘circulatory impairment’, and ‘cerebral dysfunction’ [70, pp30–1]. This purely clinical description masked experiences of distress, such as ‘uncontrollable weeping’, by medicalising them as ‘cerebral dysfunction’ [70, p31].

McEvedy and Beard’s ‘reconsideration’ of the Royal Free epidemic [54], and subsequent debate over whether ME is either a (post-)viral illness or a form of hysteria, created a deeply divided medical culture surrounding the condition. This division persists today, with physicians continuing to view ME/CFS through either biomedical or psychosomatic frameworks, despite the documented harms that psychosomatic interpretations have caused patients [71].

However, I want to emphasise that the opposing interpretations of the Royal Free epidemic came from the same ‘epistemic community’ [72], involving a network of social actors including Ramsay, McEvedy, The ME Association, *The Sunday Times, The Lancet* and *The BMJ*. On both sides of the debate, authoritative claims to knowledge were based on a ‘shared notion of validity’ [72, p3] grounded in the separation of organic and psychosomatic illness, where disease was only considered fully legitimate when proven with physical evidence. Thus, the debate between (post-)viral and hysterical framings of ME sustained, rather than challenged, medical authority. In particular, it reinforced epistemic dualism between mind and body, and in doing so entwined medical uncertainty, psychosomatic illness and femininity. For example, the *known unknown* that ME incidence was highest amongst those in feminised health professions, such as nursing, is taken as evidence for ‘epistemic hysteria’, but is ignored in biomedical explanations of disease. This legacy has meant that relations between ME and sex/gender have become ‘uncomfortable knowledge’ [17] for many ME advocates, frequently marginalised in struggles to secure recognition of ME as a legitimate illness worthy of care.

### The origins of chronic fatigue syndrome

#### Defining chronic fatigue syndrome

In 1988, the term ‘chronic fatigue syndrome’ (CFS) was introduced in the *Annals of Internal Medicine* to describe a ‘symptom complex characterized primarily by chronic or recurrent debilitating fatigue and various combinations of other symptoms’ [73, p387]. The article presented a case definition of CFS and was written by a team led by Gary Holmes and Jonathan Kaplan at the Centers for Disease Control’s (CDC) Epidemic Intelligence Service. The CDC (now the Centers for Disease Control and Prevention) is a US public health agency founded during World War II to manage outbreaks of malaria; they used insecticides as a ‘defence’ against mosquitoes and later combined this approach with disease surveillance [74]. The Epidemic Intelligence Service was established during the Cold War in response to the threat of biological warfare. It was this new program that expanded the CDC’s use of surveillance to manage public health. During the 1970s and 80s, the CDC reorganised as it shifted focus from fighting communicable diseases to dealing with more complex environmental and social challenges (such as smoking). Ironically, this changing focus was paralleled by the CDC’s new ‘war against AIDS’ [74, p340]. The CDC’s construction of CFS should be understood in the context of the HIV/AIDS epidemic, alongside their application of wartime methods of epidemiological surveillance to emerging, complex public health challenges.

The case definition of CFS [73] was presented by CDC officials as an alternative classification for a phenomenon previously referred to as ‘Epstein-Barr virus syndrome’. For the first time in the medical literature, Holmes et al. [73] linked disparate research that references forms of persistent Epstein-Barr virus (EBV) infection or chronic mononucleosis^4^, whilst simultaneously undermining its validity. Research published before the case definition associated the presence of antibodies for EBV in the blood with chronic symptoms of disabling fatigue. Some research theorised a causal relationship between EBV and symptoms [75–77], whilst other studies cited only an *association*, an unknown relationship that could be the cause or effect of disease [78]. The CDC officials also cited their previous research, which cast doubt on the relationship between EBV and chronic illness [79] and two papers that encouraged caution concerning the reliability of tests for EBV antibodies [80,81]. Holmes et al. [73] implemented a reductionist approach; they defined CFS in terms of fatigue and the exclusion of (a huge number of) other conditions, including psychiatric disease. Fatigue was framed as the hallmark of the condition, whilst all other symptoms (such as fever) were relegated to ‘minor criteria’. An article, authored by CDC officials, drew attention to a small body of literature on EBV syndrome, but redefined the condition as CFS, casting doubt on its relationship to viral infection.

#### From an epidemic to a syndrome

Holmes et al. [73], in their case definition of CFS, did not elucidate that EBV syndrome or chronic mononucleosis had been reported in clusters of the population. They omitted detail from their own reports of an outbreak of chronic mononucleosis surrounding Incline Village, Nevada [79,82], and did not acknowledge that it was this outbreak that drew the CDC’s attention to the phenomenon. The journalist Hilary Johnson [36] reconstructed events surrounding the Incline Village outbreak based on interviews with local people, physicians and health officials between 1986 and 1995. She described how, in the winter of 1984-85, in the Lake Tahoe area of Nevada notable for its wealthy residents and as a skiing destination, two internal medicine specialists, Daniel Peterson and Paul Cheney, started to notice a series of patients with unusual and disabling symptoms. By June 1985, Peterson and Cheney reported that over 90 people had been affected, including in several clusters, for example, teachers at a high school [36,83]. Peterson and Cheney identified markers of persistent EBV infection in their patients and shared their concern that an epidemic was occurring in the area with local and national health officials and the media. In September 1985, Holmes and Kaplan visited Incline Village to investigate [36,84]. Following the CDC’s visit, tensions between local physicians and public health officials were reported in the media [83,85]. In May 1986, the CDC published the results of their investigation. They selected just fifteen out of a total of 139 patients, identified ‘abnormal EBV serologic markers’, but criticised the reproducibility of the tests and their relevance to chronic illness [82]. They concluded that doctors ‘should continue to search for the more definable, and possibly treatable, conditions that may be responsible for their [patients’] symptoms’ [82].

Between 1984 and 1988, the outbreak of illness in Incline Village was transformed into CFS. From 1985 onwards, there was a ‘crescendo of media coverage’ [36, p65] of the outbreak that drew the attention of people from across the US. It was presented as a ‘medical mystery story’ in the *Los Angeles Times* [86], and patients, medical services, public health authorities and the media assembled around the controversy to vie for credibility. As emphasised by Epstein [87], these collective ‘credibility struggles’ also defined knowledge of the HIV/AIDS epidemic. The media reflected (and perpetuated) a fear that there was an outbreak akin to AIDS in the area surrounding Incline Village [88], drawing on knowledge from HIV/AIDS that a single virus could develop into a syndrome [8]. Patient groups also organised to spread awareness of the unknown disease across the nation [88], mirroring the strategies of HIV/AIDS activists [9]. Patients, doctors, the media and state institutions conducted boundary work at the ‘ragged edge of the known and the unknown’ [88], but as with the HIV/AIDS epidemic, it was state authorities that stepped in to regulate uncertainty. As Boffey wrote in the *New York Times*, the CDC and National Institutes of Health (NIH) were called upon by Congress ‘to unravel some of the mysteries’ [89, pC4]. The CDC’s reports on the outbreak in Incline Village [79,82] and the subsequent case definition for CFS [73] aimed to dampen the controversy surrounding the mystery disease. CDC officials regulated uncertainty surrounding the disease by transforming it from an unusual epidemic into a clinical syndrome, something more benign, the risk and controversy of which could be managed.

Several factors provided the conditions for this transformation from epidemic to syndrome. There were epistemic differences between the local physicians in Incline Village and the CDC officials who investigated the outbreak, including what they considered to be evidence of disease. For CDC officials, objective evidence of an outbreak of illness was determined through epidemiological methods that foregrounded reductionism and statistical significance. Whereas, for Cheney and Peterson, it was the ‘multi-system constellation’ of symptoms occurring in clusters across their local population, and the scores of patients who were distressed and disabled that made the phenomenon meaningful [36, p53]. When the Epidemic Intelligence Service at the CDC [79,82] analysed and reported on the events in Incline Village they dissolved what had been meaningful to local people about the outbreak.

The methods of the CDC that were developed to defend against threats of disease during World War II and the Cold War [74] were ill-suited to making sense of the events in Incline Village and wider concerns about EBV syndrome. CFS emerged at an inflection point for the CDC when these historic methods of disease surveillance needed to adapt to increasingly complex public health concerns, whilst the Reagan administration cut health budgets. Arguably, the epistemic differences between the local population and the CDC were also gendered; the ‘disease detectives’ at the Epidemic Intelligence Service were mostly male, ‘young, lifestyle-oriented, nonsmoking exercise buffs’ [74, p314] whose positivist methods were unable to make sense of the complex disablement of local people, the majority of whom were reportedly women [86,88]. CDC reports omitted sex and gender differences among those affected by the outbreak in Nevada; in fact, age, race and sex were essentialised and treated as confounding variables, factors that were statistically controlled in order to evaluate the relationship between EBV and illness [79,82]. This obscuring of sex and gender in medical texts has limited my analysis of the gendered characteristics of the outbreak. The reductionist epistemology of the CDC allowed CFS to be extracted from the social and environmental context of Incline Village, ultimately reducing uncertainty surrounding the disease.

The authority of the CDC’s Epidemic Intelligence Service rests on its capacity to manage public health by transforming reported epidemics into solvable problems. The CFS case definition was a public health management strategy, but the CDC managed knowledge rather than infection. Through reconstructing the Incline Village outbreak as CFS, the socio-historical origins of the illness were obscured. As with ME, uncertainty, or *known unknowns*, were masked in favour of ignorance. Looking at medical texts alone, CFS appears to be a floating concept, rising up when the previous diagnosis of neurasthenia became unfashionable, the transformation representing nothing more than the ebb and flow of medical history. The history of CFS illustrates how ignorance can be utilised as a resource to maintain authority. CFS is a form of ‘strategic ignorance’: ‘knowing the least amount possible is often the most indispensable tool for managing risks and exonerating oneself from blame in the aftermath of catastrophic events’ [19, p3].

The name and concept of ‘CFS’ was quickly able to function as an object of biomedical research and debate. Research output concerning CFS tripled in the year following the publication of the case definition. Research continued into the relationship between EBV antibodies and the condition, but adopted the new name [90–92]. Several papers were published by researchers at the NIH [93–95]. But most notable was the beginning of psychological research concerning CFS, particularly in terms of the ‘somatisation’ of psychological distress [95–97]. This research proliferated even though psychiatric disorders were excluded from the definition of CFS, i.e., according to the definition by Holmes et al. [73] CFS could not be diagnosed until psychiatric disorders were first excluded. Following the case definition, CFS became an ‘obligatory passage point’ [98] for research and debate. Whilst many people did (and continue to) oppose the CDC’s framing of CFS, it cannot be ignored completely owing to the power of the federal agency. The dampening of the historic relationship between EBV and CFS is particularly significant in light of recent research linking EBV, ME/CFS and long COVID [see e.g., 99].

## Discussion and conclusion

This analysis highlights the distinct origins of ME and CFS prior to 1990, showing how both halves of ME/CFS emerged through ‘credibility struggles’ [87], in which many actors wrestled with the boundaries of knowledge, uncertainty and ignorance. In April 1990, an international symposium on ME/CFS was held at Cambridge University, England, which brought together researchers from around the world. Following the symposium Hyde et al., published a text summarising the ‘historical, clinical, and scientific knowledge of M.E./CFS’ [34, pix]. From the 1990s onwards, ME and CFS were irreversibly entangled, as both names and concepts for framing scientific knowledge.

ME and CFS were both ways to manage uncertainty following unusual outbreaks of illness. However, they were products of distinct British and American biomedical systems, at particular historical moments. In the case of ME, uncertainty surrounding an outbreak of illness affecting mostly nurses and domestic staff at the Royal Free Hospital, London in 1955 transformed into a polarised debate as to whether the epidemic was (post-)viral or hysterical. This debate upheld the epistemic values of actors in the UK biomedical system, including a separation of mind from body, through an obscuring of ‘uncomfortable knowledge’ [17] that did not fit neatly into their paradigms. In the case of CFS, uncertainty surrounding an outbreak of illness in Incline Village, Nevada in 1984 was regulated by transforming it into the clinical case definition of CFS. The CDC’s methods of disease surveillance, premised on reductionism and statistical significance, obscured local knowledge of the epidemic. The CDC masked the social and geographic context of outbreaks, thought to be related to EBV, and in doing so created strategic forms of ignorance [19–21] that maintained their authority over US public health. The socio-historical origins of ME were largely localised around a London-centric network of patient groups, physicians, medical journals and media organisations, seemingly without the involvement of centralised public authorities. However, the roots of CFS illustrate how a national public health agency, such as the CDC, has the power to shape knowledge by institutionalising certain ways of framing disease, even in the context of the US’s geographically disparate, privatised medical system.

This analysis of the socio-historical origins of ME/CFS has provided some insight into how uncertainty, femininity and psychosomatic illness have become entangled within medical discourse surrounding the condition. Variables relating to sex and gender were largely obscured from biomedical and epidemiological research concerning the epidemics that preceded the definitions of ME and CFS. In this way, gender, and its intersections with other forms of oppression including class and race, have been made conceptually irrelevant to theories of ME and CFS as organic and legitimate. This ‘undone science’ [18] may have created a vacuum in which theories of ME/CFS as a psychosomatic disorder associated with femininity – essentially a renaming of hysteria – could persist. However, the gendered characteristics of ME/CFS cannot be examined exhaustively within the scope of this article, in particular my focus on medical documents has left me victim to the ignorance they contain. Further research involving experiential evidence is needed to understand the gendered dimensions of this history in greater depth.

The systemic production of ignorance in relation to ME/CFS is the foundation from which we should understand the epistemic injustices faced by people with the condition today. People with ME/CFS struggle for their illness to be considered serious and legitimate, and to access the healthcare and state support afforded to those who are considered credibly ill or disabled [6,22–24]. These difficulties often emerge as a moral judgement of people, predominantly women, as mentally ill and/or malingering [24]. There is a growing research area that uses the concept of epistemic injustice [26] to frame the lack of knowledge and credibility associated with ME/CFS [28–31], but researchers rarely consider the history of ME/CFS in their formulations of injustice. My analysis is a starting point for framing epistemic injustice as a structural problem, rooted in socio-historical context, rather than simply emergent from the minds and behaviours of stigmatising doctors.

This analysis illuminates uncertainty and ignorance, differing significantly from previous histories of ME/CFS that argue with certainty, on a foundation of biological essentialism, that ME/CFS is a psychosomatic illness [3,4]. I follow Aronowitz [6] in my questioning of biomedical models of ME/CFS, however, this analysis differs from his in several ways. Aronowitz [6] describes ME/CFS as emergent from a US context, tracing the origins of ME to an outbreak of illness in Los Angeles in 1934. Alternatively, my analysis has shown that ME, as a name and object of biomedical research, emerged as a response to an outbreak of illness at the Royal Free Hospital, London. The popularisation of ME, including through its connection to previous epidemics in other countries, was led by UK physicians, patient groups and media outlets. Gilliam’s [100] description of the Los Angeles epidemic in 1934 is frequently cited as the first record of an ME/CFS epidemic, however the connection between this epidemic and ME/CFS is not present in medical literature until the 1990s [see 34]. Both Aronowitz [6] and I trace the source of CFS to the Lake Tahoe region of Nevada. However, whilst he argues that CFS was popularised because of its proposed relation to a specific virus, my analysis shows that there was always uncertainty about the relationship between EBV and illness. CFS emerged as a means to deny, rather than describe the relationship between EBV and CFS. This analysis builds on previous histories of ME/CFS through an epistemological critique of biomedical frameworks and an attention to ME’s UK context.

Through an analysis of medical literature, I have traced the origins of ME and CFS to specific outbreaks of illness in the UK and the US, respectively. I have shown that whilst their origins can be rooted in particular socio-historical contexts, both ME and CFS emerged as ways to regulate biomedical uncertainty. Outbreaks of unusual, disabling symptoms of an unknown cause were transformed into clinical syndromes and scientific objects. In doing so, knowledge of these epidemics was obscured rather than generated. This production of ignorance may have limited our capacity to understand ME/CFS and created the conditions for psychosomatic research to thrive. When biomedical certainty is entangled with the legitimacy of medical conditions and the rights of people to be ill, disabled and receive care, ignorance can be favourable over acknowledging what we do not know. Instead, a pathway towards epistemic justice for people with ME/CFS may involve asserting that knowledge of suffering can be both legitimate and uncertain.

## Figures and Tables

**Table 1:** Search terms used on PubMed to identify medical literature.

Condition	Search terms
**ME**	**“myalgic encephalomyelitis”[Title]**
	“Royal Free Disease”[Title]
	neuromyasthenia[Title]
	“Iceland disease”[Title] or “Icelandic disease”[Title]
	“persistent myalgia”[Title]
	“resembling poliomyelitis”[Title]
	“simulating poliomyelitis”[Title]
	"postviral fatigue"[Title]
**CFS**	**“chronic fatigue”[Title]**
	“Epstein-Barr virus syndrome”[Title]

**Table 2:** Summary of key features of medical documents analysed.

	Type of Research			
Condition	Clinical	Epidemiological	Laboratory	Total
**ME**	24	31	1	56
**CFS**	49	9	2	60
**ME/CFS**	2	0	0	2
Total	75	40	3	118

## Data Availability

Data sharing is not applicable as no data sets were generated and/or analysed for this study.

## Footnotes

1 PEM is understood as an exacerbation of symptoms after physical or mental activity, the onset of which can be delayed by hours or days.

2 **Patient and public involvement**. Patients and the public were not involved in this analysis. This research is part of an ethnographic project, involving patients, clinicians and researchers in both its design and dissemination.

3 One patient died by suicide following the epidemic.

4 Mononucleosis, often referred to as ‘mono’ or ‘glandular fever’ results from infection with Epstein-Barr virus.
